# Action of Natural Products on P2 Receptors: A Reinvented Era for Drug Discovery

**DOI:** 10.3390/molecules171113009

**Published:** 2012-11-01

**Authors:** Robson Faria, Leonardo Ferreira, Rômulo Bezerra, Valber Frutuoso, Luiz Alves

**Affiliations:** 1 Laboratory of Cellular Communication, Oswaldo Cruz Foundation, 4365 Rio de Janeiro, Brazil; Email: robson.xavier@gmail.com (R.F.); leonardobraga12@gmail.com (L.F.); romulo@ioc.fiocruz.br (R.B.); 2 Laboratory of Immnopharmacology, Oswaldo Cruz Foundation, 4365 Rio de Janeiro, Brazil; Email: frutuoso@ioc.fiocruz.br

**Keywords:** P2 receptors, natural products, antagonists

## Abstract

Natural products contribute significantly to available drug therapies and have been a rich source for scientific investigation. In general, due to their low cost and traditional use in some cultures, they are an object of growing interest as alternatives to synthetic drugs. With several diseases such as cancer, and inflammatory and neuropathic diseases having been linked to the participation of purinergic (P2) receptors, there has been a flurry of investigations on ligands within natural products. Thirty-four different sources of these compounds have been found so far, that have shown either agonistic or antagonistic effects on P2 receptors. Of those, nine different plant sources demonstrated effects on P2X2, P2X3, P2X7, and possibly P2Y12 receptor subtypes. Microorganisms, which represent the largest group, with 26 different sources, showed effects on both receptor subtypes, ranging from P2X1 to P2X4 and P2X7, and P2Y1, P2Y2, P2Y4, and P2Y6. In addition, there were seventeen animal sources that affected P2X7 and P2Y1 and P2Y12 receptors. Natural products have provided some fascinating new mechanisms and sources to better understand the P2 receptor antagonism. Moreover, current investigations should clarify further pharmacological mechanisms in order to consider these products as potential new medicines.

## 1. Introduction

Natural products have long been used in traditional Western and Eastern medicine, and they are being actively pursued for drug therapies today. Natural products, which are derived not only from plants, but also from fungi, bacteria, and marine organisms, present distinctive characteristics in their secondary metabolites [1]. The unique properties of these secondary metabolites are often involved in defense and interaction with the environment, and as such they have often given rise to extraordinarily useful drugs, such as penicillin and morphine. These secondary metabolites, which the body can properly digest and process, have been and will likely continue to be, a rich source of alternatives to synthetic drugs [2]. Several factors have facilitated the search for natural products as potential new drug therapies. These factors include, but are not limited to, isolation techniques, such as spectroscopic, chromatographic, biosynthetic, and synthetic methods, and the fact that these isolates can now be obtained by synthetic or combinatorial chemistry and molecular modeling [3,4,5]. These advances have allowed for the investigation of natural products to become much easier and more time efficient as a source of therapeutic strategies.

One relevant area of investigation is the group of ionotropic (P2X) and metabotropic (P2Y) receptors, which have been found in all studied cells so far. These purinergic receptors, also known as P2 receptors, are crucial for the normal function of an organism, and they have been associated with some disease processes, such as rheumatoid arthritis [6], pain [7,8], and cancer [9] development. In the search for understanding the role of P2 receptors in these diseases, selective agonistic and antagonistic ligands have been designed and used as therapeutic agents and pharmacological tools [10]. In this context, many effective ligands have been found, but selective targeting of some receptor subtypes still remains elusive. This is the case for P2Y4, P2X2, and P2X5 receptors, where no such ligands have been discovered. This article focuses on some of the basic properties of these P2 receptors and the natural products that act on them as either agonists or antagonists. Herein, we discuss and give evidence of the large potential of natural products as possible modulators of P2X and P2Y receptors.

## 2. The P2—ATP Activated Receptors

Since the 1970s, it has been clearly demonstrated that ATP and other analogues are extracellular messengers, acting on purinergic receptors. ATP can be released without cell lysis by specific mechanisms such as: exocytosis, ABC transporters, and membrane channels (e.g., pannexins, connexins, maxi-anion channel, and others) [11,12]. Once in the extracellular fluid, nucleotides can activate the P2X and P2Y families of purinergic receptors. The P2X group comprises of seven inotropic receptors found in mammalian cells: P2X1, P2X2, P2X3, P2X4, P2X5, P2X6, and P2X7. The P2Y family is composed of metabotropic receptors, having eight subtypes in mammalian cells: P2Y1, P2Y2, P2Y4, P2Y6, P2Y11, P2Y12, P2Y13, and P2Y14. The P2Y1, P2Y2, P2Y4, P2Y6, and P2Y11 receptors are coupled to Gq, activating phospholipase C-β. The P2Y12, P2Y13, and P2Y14 receptors are coupled to Gi, inhibiting adenylyl cyclase. The P2Y11 receptor has the unique property of coupling with both Gq and Gs.

In general, the P2X receptors are more structurally restrictive than P2Y in agonist selectivity. There is a cross-reactivity of P2Y receptor probes with P2X receptors, meaning that some of these probes are not altogether selective. More specifically, ATP acts as the active ligand for P2X receptors, whereas in addition to ATP, P2Y receptors respond to naturally occurring nucleotides such as UDP, ADP, UTP, and UDP glucose. Among P2X receptors, subtypes P2X2, P2X4, and P2X7, in the presence of millimolar ATP concentrations and prolonged activation time, form a nonselective pore with a cut of up to 900 Da, depending on cellular type and specie analyzed [13]. So far, it is an open question whether these ion channels dilate or activate another protein responsible for high conductance channels to allow the passage of small molecules, such as Lucifer yellow, Yo-pro, and propidium iodide. Up until now, there have not been specific compounds to discriminate between low and high conductance channels [13,14].

So far, less than one hundred natural products have been identified that act on P2 receptors. They are divided by species and classified as being of animal, plant, or microorganism origin as described in next sections.

## 3. Natural Products from Plant Sources Acting on P2 Receptors

Among all types of natural products, plant extracts have received the greatest attention until now. In this section, we describe and discuss plant extracts that act by modulating the activity of P2X and P2Y receptors. As can be seen in Table 1, there are three purified compounds and seven crude extracts derived from from plants. As their number is small, we will provide a more detailed description of their effects.

**Table 1:** Natural products from plant sources.

Compound	Receptor Type	Effects (IC_50_/EC_50_) *	Tested Model	Reference
Mustard oil	P2X3	Participation in sensitization in nociceptive neurons following MO application to the tooth pulp. ND	Male Sprague-Dawley adult rats	[15]
Sodium Ferulate	P2X3	Decreases participation of these receptors in pain after primary sensory afferent chronic injury ND	Rat dorsal root ganglion	[16]
Tetramethylpyrazine	P2X3	Inhibition of depolarization, burn injury pain and neuropathic pain induced by α,β-methylene-ATP ND	Rat dorsal root ganglion	[17,18,19,20]
Puerarin	P2X3	Impairment of neuropathic pain ND	Dorsal root ganglion neurons	[21,22]
Emodin	P2X2/3	Inhibition of the transmission of neuropathic pain stimuli ND	Sprague-Dawley male rats	[23]
Emodin	P2X7	Inhibits ATP/BzATP-activated P2X7 receptor IC_50_ = 200 nM (cell death)	Rat peritoneal macrophages	[24]
Emodin	P2X7	Inhibits ATP/BzATP-activated P2X7 receptor IC_50_ = 500 nM (BzATP- and induced dye uptake)	Rat peritoneal macrophages	[24]
Emodin	P2X7	Inhibits ATP/BzATP-activated P2X7 receptor IC_50_ = 3.4 µM (BzATP-evoked current)	HEK 293	[24]
*Rheedia.longifolia* methanol extract	P2X7	Inhibits P2X7 receptor-associated pore opening, currents and dye uptake functional assay IC_50_ = 2 µg/mL (functional assay)	Mouse peritoneal macrophages	[25]
Flavonoid molecules	P2Y2	Potent antagonism and inhibition of intracellular calcium release ND	NG108-15 cells	[26]
*Trigonella foenum* leaf extract	P2Y12 (?)	Inhibition of ADP-induced platelets aggregation IC_50_ = 1.28 mg/mL	Rabbit platelets	[27]
*Trigonella foenum* leaf extract	P2X	Inhibits α,β-methylene-ATP (30 µM) induced isometric contraction IC_50_ = 1.57 mg/mL	Mouse vas deferens	[27]
Colchicine	P2X7	Inhibits P2X7 receptor-associated pore opening EC_50_ = 290 μM	Xenopus laevis oocytes	[28]
Colchicine	P2X7	Inhibits P2X7 receptor-associated pore opening EC_50_ = 540 μM	Peritoneal mouse macrophages	[28]

* EC_50_ = half maximal effective concentration; IC_50_ = half maximal inhibitory concentration; ND = Not Determinated; MO = Mustard oil; BzATP = 3'-*O*-(4-benzoyl)benzoyl adenosine 5'-triphosphate.

P2X2/3 (heterometric receptor) and P2X3 receptors in trigeminal subnucleus caudalis are involved in the initiation and maintenance of central sensitization in subnucleus oralis nociceptive neurons induced by mustard oil application to the tooth pulp in anesthetized rats, this effect is possible associated with the neuroplastic changes in receptors NMDA (*N*-methyl-D-aspartate receptors) [15]. In chronic pain mediated by P2X3 receptors, sodium ferulate, an active principle from Chinese herbal medicine with anti-inflammatory activities, inhibited the nociceptive facilitation of the primary sensory afferent neurons after chronic constriction injury [16,29]. In this context, there are several papers describing the effects of Chinese herbal medicines on P2X3 and P2X2/3 receptors on reducing pain. The ligustrazine alkaloid tetramethylpyrazine has been studied with analgesic purposes in the context of nociceptive responses [17,18], burn injury pain [19], and neuropathic pain [20] induced by α,β-methylene-ATP. The burn injury pain transmission mediated by P2X3 receptor may be reduced by puerarin, which is one of the three major isoflavonoid compounds and has been widely used in treatment of myocardial and cerebral ischemia [30]. Puerarin may also impair the neuropathic pain mediated by P2X3 receptor in dorsal root ganglion neurons [22].

In 2004, Shemon observed that chelerythrine, a benzophenanthridine alkaloid, blocks the ATP- induced cation fluxes mediated by the P2X7 receptor, as well as the ATP induced stimulation of phospholipase D in human B lymphocytes [31]. Said, in 2007, described the *in vitro* and *in vivo* toxicity of four vegetable oils compared to castor oil to validate their use as vehicles of lipophilic drugs in eye drops [32]. P2X7 receptor activation was a parameter analyzed to assess the cytotoxicity induced by these oils. They observed that only castor oil promoted P2X7 receptor activation. In addition, Coutinho-Silva’s group investigated the effect of mineral oil and thioglycolate, substances capable of recruiting macrophages into mouse peritoneal cavity, on P2X7 receptor expression and function. They found that mineral oil induced P2X7 down regulation associated with reduced functional activity of this receptor [33]. In the paper published by Liu and colleagues in 2010, the authors studied the anti-inflammatory and immunosuppressive mechanisms of emodin (1,3,8-trihydroxy-6-methylanthraquinone), an anthraquinone derivative from *Rheum officinale Baill*. The P2X7 receptor activities induced by ATP or BzATP were inhibited by emodin pre-treatment in native macrophages or transfected HEK-293 cells with P2X7R [24]. Interestingly, emodin was able to impair P2X2/3 receptor role in transmission of neuropathic pain stimuli of primary sensory neurons in Sprague-Dawley rats [23]. Santos and coworkers have demonstrated that the extract and fractions of *Rheedia longifolia* inhibited the P2X7 receptor-induced dye uptake and ionic currents. After chromatography analysis, they identified the bisflavonoids as the most probable active compounds responsible for P2X7 inhibitory effects present in the *R. longifolia* extract and fractions [25].

The *Colchicum* sp*.* secondary metabolite colchicine is traditionally associated with gout treatment because of its ability to disturb cytoskeletal microfilaments, inhibiting inflammatory cell activation. Marques-da-Silva and colleagues showed that *in vitro* dolchicine was able to inhibit pore opening, but not the P2X7 receptor low-conductance channel. Furthermore, dolchicine also diminished the maturation and release of IL-1β and production of nitric oxide and reactive oxygen species induced by ATP. Interestingly, these effects were specific to dolchicine and were not found with other mitotic inhibitors, such as taxol and vincristine [28].

In relation to P2Y receptors, Mendes and colleagues, in 2003, observed P2Y1 or P2Y2 receptors’ participation in the mechanism of the vascular relaxation produced by polyphenolic substances from red wine [34]. Kaulich and colleagues evaluated a series of 40 flavonoids as antagonists at P2Y2 receptors expressed in NG108-15 cells. By measuring the inhibition of UTP-stimulated intracellular calcium release, they identified diverse flavonoids as potent antagonists at P2Y2 receptors, with IC_50_ values in the low micromolar range and potency similar or higher than the standard P2Y2 antagonists Reactive Blue 2 and Suramin [26]. Polyphenolic compounds extracted from *Aronia melanocarpa* fruits have been reported to be cardioprotective agents. The Luzak group examined the ability of *Aronia melanocarpa* extract to increase the efficacy of human umbilical vein endothelial cells to inhibit platelet functions *in vitro*. They observed that only at low concentrations (5 µg/mL) did *Aronia melanocarpa* extract significantly improve antiplatelet action of human umbilical vein endothelial cells towards ADP-activated platelets in the aggregation test [35]. In another work, ADP-activated platelet aggregation was inhibited by ethyl acetate extract from *Opuntia humifusa raf*. This extract inhibited ADP-induced intracellular calcium mobilization and ATP release [36].

Parvizpur and collaborators observed that *Trigonella foenum* (TFG) leaf extract can exert analgesic effects in both formalin and tail flick tests [37,38]. In another paper [27], they studied the involvement of purinergic receptors in the formalin and tail flick tests. The TFG extract [0.5, 1, 1.5, 3 mg/mL] inhibited ADP [10^−5^ mol] induced platelet aggregation [IC_50_ = 1.28 mg/mL]. α,β-methylene-ATP [30 mM] induced isometric contraction in the vas deferens was inhibited by Suramin, a P2 receptor antagonist or TFG extract [IC_50_ were 91.07 μM and 1.57 mg/mL, respectively].

## 4. Natural Products from Animal Sources Acting on P2 Receptors

Animal sources of natural products have received less attention in the search for discovering new medicines. Nevertheless, new medicines, most of which are of invertebrate origin, have been approved for human use. According to Table 2, there are sixteen purified compounds from animal origin, which are discussed below.

**Table 2:** Natural products from animal sources.

Compound	Receptor Type	Source	Effects (IC_50_/EC_50_) *	Tested Model	Reference
Halistanol sulfate	P2Y12	*Topsentia* sp*.*	Binds to P2Y12 receptor IC_50_ = 0.48 µM	1321N cells	[39]
Sterol sulfate Sch 572423			Binds to P2Y12 receptor IC_50_ = 2.2 µM	1321N cells	[39]
Iso-iantheran-A	P2Y11	*Ianthella quadrangulata*	Activates P2Y11 receptor EC_50_ = 1.29 µM	1321N1 wild-type cells	[40]
Iso-iantheran-B	P2Y11	*Ianthella quadrangulata*	Activates P2Y11 receptor EC_50_ = 0.48 µM	1321N1 wild-type cells	[40]
Stylissadines A	P2X7	*Stylissa flabellata*	Inhibits BzATP-induced pore formation IC_50_ = 0.7 µM	THP-1 cells. (Human Monocytes)	[41]
Stylissadines B			Inhibits BzATP-induced pore formation IC_50_ = 1.8 µM	THP-1 cells	[41]
Niphatoxin C	P2X7	*Callyspongia* sp.	Impairment of P2X7 receptor activity ND	THP-1 cells	[42]
LL37	P2X7	Human neutrophils and epithelial cells	Induces IL-1β maturation and release in LPS-primed monocytes ND	Human monocytes	[43]
rCRAMP	P2Y	*Ratus novergicus*	Induction of IL-6 expression and ERK 1/2 in glial cells, blocked by P2Y receptor antagonists ND	Rat glial cells	[44]
CRAMP	P2X7	*Mus musculus*	Inhibition of all responses related to P2X7 activation ND	Peritoneal macrophages	[45]
Cellular prion protein	P2X4	*Homo sapiens*	Prevents and reverses Copper-inhibited ATP-evoked current EC_50_ = 4.6 µM	*Xenopus laevis* oocyte	[46]
Melittin	P2X2/3 and P2X3	Apitoxin (bee venom)	Antagonists of both receptor suppressed melittin-evoked persistent spontaneous nociception ND	Male Sprague-Dawley albino rats weighing 180–250 g	[47]
Alphadefensin 1–3	P2Y6	Human CD14^−^/CD24^+^ cells	Inhibits M-CSF-induced differentiation of CD14^−^/CD24^+^ cells through P2Y6 receptor ND	CD14^+^/CD24^−^ monocytes human cells	[48]
Ω-Conotoxin GVIA	P2X2/3	*Conus* sp.	Inhibits P2X2/3 receptor response IC_50_ = 3.84 µM	Rat dorsal root ganglion neurons	[49]
	P2X3		Inhibits P2X3 receptor response IC_50_ = 21.2 nM	Rat dorsal root ganglion neurons	[49]
Purotoxin-1	P2X	*Lycosa* spider	Inhibition of ionic currents in the sensory neurons of rats ND	Rat dorsal root ganglion neurons	[50]
Purotoxin-1	P2X3	*Geolycosa* sp.spider venom	Potent inhibitory effects ND	Sensory neurons	[51]

* EC_50_ = half maximal effective concentration; IC_50_ = half maximal inhibitory concentration; ND = Not Determinated.

Several research groups consider marine organisms as a reliable source of new drugs. On par with this idea, a diverse array of compounds obtained from marine animals are under clinical trials [52]. Bioassay-guided fractionation of an active fraction of the marine sponge *Topsentia* sp. (*Halichondriidae*) obtained from a marine fraction library led to the isolation and identification of halistanol sulfate and of a new sterol sulfate compound, denominated Sch 572423. Both compounds inhibited the P2Y12 receptor [39]. Greve and collaborators isolated three new iantherans (iso-iantheran A, 8-carboxy-iso-iantheran A, and iso-iantheran B) composed of a rare dimeric benzofuran skeleton, including a 2,3-dihydroxy-1,3-butadiene disulfate moiety, obtained from the marine sponge, *Ianthella quadrangulata*. Biological assays demonstrated an agonist effect of iso-iantheran-A and iso-iantheran-B on P2Y11 receptors with EC_50_ values of 1.29 µM and 0.48 µM, respectively [40].

In 2007, Buchanan and colleagues [41] published three papers on P2X7 receptor function, based on a natural product high throughput screening effort to discover selective P2X7 receptor antagonists using marine sponge derivatives. Initially, they observed that the Australian marine sponge *Stylissa flabellata* was related to the blockage on the cationic current trough the P2X7 receptors by benzophrenatridins alkaloids (stylissadines A and B). This action was not selective however, and they noted the inhibition of some enzymes by this phytochemistry compound, such as protein kinase C and alanine aminotransferase. Both compounds inhibited P2X7 receptor function with IC_50_ values of 0.7 µM and 1.8 µM, respectively [41]. In another paper, these authors used the Australian marine sponge *Callyspongia* *sp*. (*Callyspongiidae*) and isolated the bioactive constituents the Niphatoxin C, which belongs to the 3-alkylpyridinium class of alkaloids. This constituent impaired the P2X7 receptor activity on THP-1 cells [42].

Melittin is the principal active component of apitoxin (bee venom) and is a powerful stimulator of phospholipase A2. The subcutaneous injection of melittin could induce persistent spontaneous nociception and primary thermal or mechanical hyperalgesia, but the exact peripheral mechanisms remain unclear. Post-treatment of the primary injury site with subcutaneous injection of A-317491 (P2X3 and P2X2/3 receptor antagonist) and Reactive Blue 2 (general P2Y receptor antagonist) suppressed the melittin-evoked persistent spontaneous nociception and hypersensitivity (thermal and mechanical) responses [47].

Grishin and colleagues demonstrated that the spider venom purotoxin-1 can inhibit P2X3 receptor function and delay the recovery rate from its desensitization [51]. Moreover, this 35-amino acid single-chain peptide also down regulates primary afferent sensory neurons leading to antinociceptive states with attractive 12 nM concentration. It is noteworthy that the concentration of purotoxin-1 was 3-fold lower than the P2X3 and P2X2/3 receptor antagonist A-317491, which encourages development of novel pain killer drugs. In 2009, Savchenko and colleagues studied the modulatory effect of peptide compounds of *Lycosa* spider venom on the ionic currents in the sensory neurons of rats through P2X receptors in rat dorsal root ganglion neurons [50].

Antimicrobial peptides (also called host defense peptides) are an evolutionarily conserved component of the innate immune response and are found among all classes of organisms. In general, these peptides are potent, broad-spectrum antibiotics, but in some cases, as we describe below, these peptides may modulate the ionic channels.

The human cathelicidin-derived peptide LL37 is a potent antimicrobial peptide produced predominantly by neutrophils and epithelial cells. LPS-primed monocytes stimulated with LL37 lead to the maturation and release of interleukin-1beta (IL-1beta) via the P2X7 receptor. IL-1beta release and cell permeability were suppressed by pretreatment with the P2X7 receptor inhibitors oxidized ATP, KN04, and KN62 [43]. LPS-primed monocytes, stimulated with LL37, resulted in P2X7 receptor maturation and release of IL-beta.

Another peptide, the antibacterial cathelicidin rCRAMP (homologue of the human LL-37), not only exhibits potent bactericidal activities in rats, but also functions as a chemoattractant for immune cells. Brandenburg and colleagues [44] showed that rCRAMP-induced IL-6 expression and ERK1/2 phosphorylation in glial cells. This effect might be mediated by P2Y11 and was not mediated by P2X receptors since those that block the P2X receptors did not affect the production of IL-6. On the other hand, Seil and collaborates described that CRAMP, also in mice, inhibited all the responses coupled to P2X7 receptors in macrophages [45].

Another family of peptides denominated as conotoxins, which belong to a group of neurotoxic peptides isolated from the venom of the marine cone snail, genus *Conus*, was found to modulate several types of ionic channels, including N-type calcium channels, which selectively act in the ascending pain pathway [53,54]. Extracted from *Conus* snails*,* Ω-conotoxin GVIA inhibits P2X3 and P2X2/3 receptor-mediated responses with IC_50_ values of 21.2 nM and 3.84 µM, respectively [49].

The human alpha-defensin 1–3, which is a small arginine-rich peptide, participates in the host immune defense, and is secreted by CD14^−^/CD24^+^ cells. This peptide has been shown to inhibit macrophage-colony stimulating factor induced differentiation of CD14^−^/CD24^+^ cells, at least in part through P2Y6, a receptor involved in macrophage differentiation [48].

Cellular prion protein physiological function remains unknown, but there is evidence supporting its role in copper homeostasis. Lorca and his group have shown that the perfusion of this domain prevents and reverses the inhibition by Cu^2+^ of ATP-evoked currents of the P2X4 receptor subtype, highlighting a modulatory role for cellular prion protein in synaptic transmission through regulation of Cu^2+^ levels [46].

## 5. Natural Products from Microorganisms Acting on P2 Receptors

Substances produced by microorganisms that modulate tissues, cell types, and protein function, have long been known as shown in Table 3. To this effect, ivermectin, a semisynthetic derivative of the natural fermentation products of *Streptomyces avermitilis*, is widely used in human and veterinary medicine as an antiparasitic agent [55]. In 1999, Khakh showed that ivermectin is a specific positive allosteric effector of heterologously expressed P2X4, but not of P2X2, P2X3, P2X2/3, or P2X7 receptor channels in rats [56]. This result was confirmed by other groups in humans [57], mice [58], and rats [59]. Recently, however, Nörenberg *et al*. 2012 [60] described a positive allosteric effector of ivermectin also on human P2X7 receptor.

The natural peptide polymyxin B is a well-known and potent antibiotic that binds and neutralizes bacterial endotoxin lipopolysaccharide. Ferrari demonstrated that polymyxin B increased the responses mediated by the P2X7 receptor in HEK293 and K562 cell lines transfected with P2X7 receptor cDNA, as well as in mouse and human macrophages [61].

**Table 3:** Natural products from microorganism product sources.

Compound	Receptor Type	Source	Effects (IC_50_/EC_50_) *	Tested Model	Reference
Ivermectin	P2X4	*Streptomyces avermitilis*	Positive allosteric effect EC_50_ = 250 nM	*Xenopus laevis* oocyte	[56]
Ivermectin	P2X4	*Streptomyces avermitilis*	Blockage of ethanol-inhibitory effects ND	*Xenopus laevis* oocyte	[62]
Ivermectin	P2X7	*Streptomyces avermitilis*	Positive allosteric effect EC_50_ = 50 nM (EC_50_ from high affinity-binding site)	Macrophage from humans	[60]
Polymyxin B	P2X7	*Bacillus polymyxa*	Enhanced P2X7 responses in transfected-HEK293 and K562 cells ND	Mouse and human macrophage cells	[61]
Pfiesteriatoxin	P2X7	*Pfiesteriapiscicida*	Activation of cell permeabilization similarly to ATP activation ND	GH4C1 rat pituitary cells	[63]
Pfiesteriatoxin	P2X7	*Pfiesteriapiscicida*	Induction of toxic and c-*fos* luciferase that is blocked by oxATP and PPADS ND	GH4C1 rat pituitary cells	[64]
HlyA	P2X1 and P2X7	*Escherichia coli*	Antagonists of both receptor blocked HlyA induced hemolysis ND	Human, mouse and equine Erythrocytes	[65]
Cytotoxic factors	P2X7	*Pseudomonas aeruginosastrain 808*	P2X7 receptor participation in ATP-dependent pathway ND	J774 macrophage cell line	[66]
Leukotoxin	P2X7	*Aggregatibacter actinomycetemcomitans*	Leukotoxin-induced proinflammatory responses, release of IL-1β and IL-18 are blocked by oxATP ND	Human macrophages	[67]
Oxo-AHL	P2Y2 and P2Y4	*Pseudomonasaeruginosa*	Inhibits P2Y2 and P2Y4 expression in cystic fibrosis IC _50_ = 0.3 pM	HTGS cell line MM39	[68]
LPS	P2X7	Gram-negative bacteria	P2X7 receptor modulates LPS-induced responses ND	Murine Peritoneal Macrophages	[69]
LPS	P2X7	Gram-negative bacteria	P2X7 receptor inhibition in TLR-4-defficient cell ND	HEK293 cells	[70]
LPS	P2Y6	Gram-negative bacteria	Vascular inflammation following selective induction of endothelial P2Y6 receptor ND	HMEC-1	[71]
LOS	P2X	Gram-negative bacteria	Inhibition of P2X receptor decrease LOS-induced caspase-8 activation and apoptosis ND	Primary bovine pulmonary artery endothelial cells	[72]

* EC_50_ = half maximal effective concentration; IC_50_ = half maximal inhibitory concentration; ND = Not Determinated; LOS = Lipooligosaccharide.

*Pfiesteria piscicida* is a dinoflagellate that produces the putative bioactive substance *Pfiesteria* toxin, which displays toxicity in fishes and humans. This substance induced cell permeabilization in a similar manner as ATP in culture of GH4C1 rat pituitary cells expressing functional P2X7 receptors. This effect was inhibited by the P2X7 receptor antagonist oxidized ATP [63]. In addition, Kimm-Brinson examined the pharmacological activity of the *Pfiesteria* toxin on the signaling pathway that induces the *c-fos* luciferase construct in GH4C1 rat pituitary cells. ATP-, BZATP-, and *Pfiesteria* toxin-induced cytotoxicity, and *c-fos* luciferase activity was inhibited by pyridoxalphosphate-6-azophenyl-2',4'-disulfonic acid and the P2X7 irreversible antagonist oxidized-ATP [64].

*Escherichia coli*, which exhibits facultative and invasive strains, is the dominant facultative bacterium in the normal intestinal flora, but it is also responsible for the majority of serious extraintestinal infections. Alpha-hemolysin (HlyA) is a virulence factor produced by invasive *E. coli* strains, which causes hemolysis by forming pores in the erythrocyte membrane and triggers purinergic receptor activation to mediate the full hemolytic action. General P2 antagonists (pyridoxalphosphate-6-azophenyl-2',4'-disulfonic acid and suramin) and ATP scavengers (apyrase, hexokinase) inhibited HlyA-induced lysis of equine, murine, and human erythrocytes. P2X1 and P2X7 receptors antagonists indicated both receptors to be involved in the HlyA-induced hemolysis [65].

Cytotoxic factors released by a nonmucoid clinical isolate of *Pseudomonas aeruginosa*, strain 808, produced ATP-dependent and -independent responses. The ATP dependent pathway utilizes the P2X7 receptor to exert its effects, in contrast to the ATP-independent pathway [66].

*Aggregatibacter (Actinobacillus) actinomycetemcomitans* is a facultative anaerobic Gram-negative bacterium associated with severe forms of periodontitis and several virulence factors. Among those, the leukotoxin is suggested to have an important role in its pathogenicity [73,74]. This toxin is able to lyse human immune cells and induces significant secretion of the pro-inflamatory cytokines IL-1 and IL-18 in human macrophages [67,75]. This pro-inflammatory response was inhibited by oxidized ATP, which indicates the involvement of the P2X7 receptor [67].

Cystic fibrosis is a genetic disease characterized by the hypersecretion of mucus, inflammation in the airways, and especially by persistent severe bacterial infections, generally by the gram-negative bacterium *Pseudomonas aeruginosa* [76]. ATP or UTP analogues were shown to induce chloride secretion by cystic fibrosis epithelial cells [77] and also to induce bronchial relaxation [78]. *P. aeruginosa* virulence factors can be regulated by two unique quorum sensing systems [79] composed by a small diffusible signal molecule (*N*-acylhomoserine lactone [AHL]) and a transcriptional activator protein [80]. In this work, the authors tested the effects of AHLs on HTGS cells regarding its action upon P2 receptors. Oxo-AHLs treatments repressed the stimulatory effects of secretory leukocyte proteinase inhibitor secretion by nucleotides, possibly due to the repression of P2Y2 and P2Y4 receptor expression, in cystic fibrosis but not normal HTGS cells [68].

Components of gram-negative bacteria have been studied in the context of the purinergic signaling in inflammation processes that promote an increase of nucleotide concentrations. The bacterial endotoxin LPS is one of the strongest stimuli for the immune response, and several papers have shown that P2X7 receptor activation can modulate LPS-induced responses [69,81]. Interestingly, a binding site for LPS within the P2X7 receptor C-terminus has been proposed. Accordingly, Leiva-Salcedo and colleagues investigated if LPS can directly modulate the activity of the P2X7 receptor. They found that LPS alone was unable to induce any P2X7 receptor-related activity, suggesting that the receptor is not directly activated by the endotoxin. On the other hand, pre-application of LPS inhibited P2X7 receptor ionic channel and pore function in HEK293 cells [70]. Compellingly, another bacterial product lipooligosacharide also is able to promote the release of ATP and its derivatives, which can enhance the processes of cell activation and apoptosis. These lipid A-containing compounds stimulate endothelial cells to take part in several inflammatory steps, such as production and release of reactive oxygen species, cytokine, and again, the “universal” danger-associated molecule pattern, ATP [72].

Some microorganisms, such as yeast and bacteria, are capable of producing alcohols; as such, the effect of short alcohols was described [82,83]. ATP-activated ionic currents in presynaptic and postsynaptic membranes, due to P2X4 and P2X2 receptor activation, were reduced by ethanol treatment [83]. In 2011, Ostrovskaya demonstrated that ethanol inhibits ATP-gated currents mediated by P2X4 receptor in a rapid manner, and the inhibition does not depend on voltage or ATP concentration [84].

In another paper, Asatryan showed that ivermectin may reverse the inhibitory effects of ethanol in P2X4 receptor in dose-dependent manner [62]. Fischer and collaborates demonstrated that P2X3, a receptor widely expressed in dorsal root ganglion neurons, seems to be distinctly modulated by 2,2,2-thicloroethanol. As compared to ethanol, 2,2,2-thicloroethanol inhibited current responses and intracellular calcium elevation of ATP and its analogue α,β-methylene-ATP [85]. Davies, in 2005, transfected the hP2X3 and hP2X4 receptors to *Xenopus* oocytes and studied the ionic current after ATP treatment. Ethanol reduced P2X4 receptor responses and increased the maximal response of P2X3 receptor ionic currents [86].

During a systemic inflammatory response, endothelial-expressed surface molecules have been strongly implicated in regulating immune responses. Based on previous studies about exhibiting enhanced extracellular nucleotide released during acute inflammation, Riegel postulated that endothelial nucleotide receptors could play a role in vascular inflammation. Pharmacologic or genetic *in vivo* studies showed attenuated inflammatory responses in *P2Y6*^−*/*−^ mice or after P2Y6 antagonist treatment during LPS-induced vascular inflammation [71].

## 6. Conclusions

The association of diseases with expression of purinergic receptors should prompt further search for pharmacological compounds with selective action on P2X and P2Y receptors. Accordingly, the results described here revealed that several promising natural products present significant agonist or antagonist activities for P2 receptors. A better understanding of the role of P2 receptors in physiological and pathological processes will be a key element in the discovery of new medicines for several pathological conditions such as cancer, rheumatoid arthritis, and pain.
